# Extracellular Vesicles: The Landscape in the Progression, Diagnosis, and Treatment of Triple-Negative Breast Cancer

**DOI:** 10.3389/fcell.2022.842898

**Published:** 2022-03-01

**Authors:** Menglu Dong, Quan Liu, Yi Xu, Qi Zhang

**Affiliations:** ^1^ Department of Thyroid and Breast Surgery, Tongji Hospital, Tongji Medical College, Huazhong University of Science and Technology, Wuhan, China; ^2^ Department of Thyroid and Breast Surgery, Xiantao First People’s Hospital Affiliated to Yangtze University, Xiantao, China; ^3^ Department of Plastic and Cosmetic Surgery, Tongji Hospital, Tongji Medical College, Huazhong University of Science and Technology, Wuhan, China

**Keywords:** triple-negative breast cancer, extracellular vesicle, metastasis, diagnosis, carriers

## Abstract

Triple-negative breast cancer (TNBC) is a heterogeneous subtype of breast cancer (BC) with diverse biological behavior, high aggressiveness, and poor prognosis. Extracellular vesicles (EVs) are nano-sized membrane-bound vesicles secreted by nearly all cells, and are involved in physiological and pathological processes. EVs deliver multiple functional cargos into the extracellular space, including proteins, lipids, mRNAs, non-coding RNAs (ncRNAs), and DNA fragments. Emerging evidence confirms that EVs enable pro-oncogenic secretome delivering and trafficking for long-distance cell-to-cell communication in shaping the tumor microenvironment (TME). The transferred tumor-derived EVs modify the capability of invasive behavior and organ-specific metastasis in recipient cells. In addition, TNBC cell-derived EVs have been extensively investigated due to their promising potential as valuable biomarkers for diagnosis, monitoring, and treatment evaluation. Here, the present review will discuss the recent progress of EVs in TNBC growth, metastasis, immune regulation, as well as the potential in TNBC diagnosis and treatment application, hoping to decipher the advantages and challenges of EVs for combating TNBC.

## 1 Introduction

Breast cancer (BC) is the leading cause of cancer-associated mortality among women worldwide (Sung et al., 2021). Triple-negative breast cancer (TNBC) is a dangerous BC subtype that lacks three widely used diagnostic markers, including human epidermal growth factor receptor 2 (HER-2), progesterone receptor (PR), and estrogen receptor (ER), accounting for approximately 15%–20% of BC presentations (Jiang et al., 2021). TNBC is particularly characterized by poor prognosis, high aggressiveness, high mortality rate, and shorter median time to relapse. TNBC is more prone to colonizing the lungs, liver, and brain and is often unresponsive to targeted therapies (Bianchini et al., 2021). Therefore, the in-depth exploration of molecular mechanisms and targeted therapies of refractory metastatic TNBC is a clinical imperative.

**FIGURE 1:**
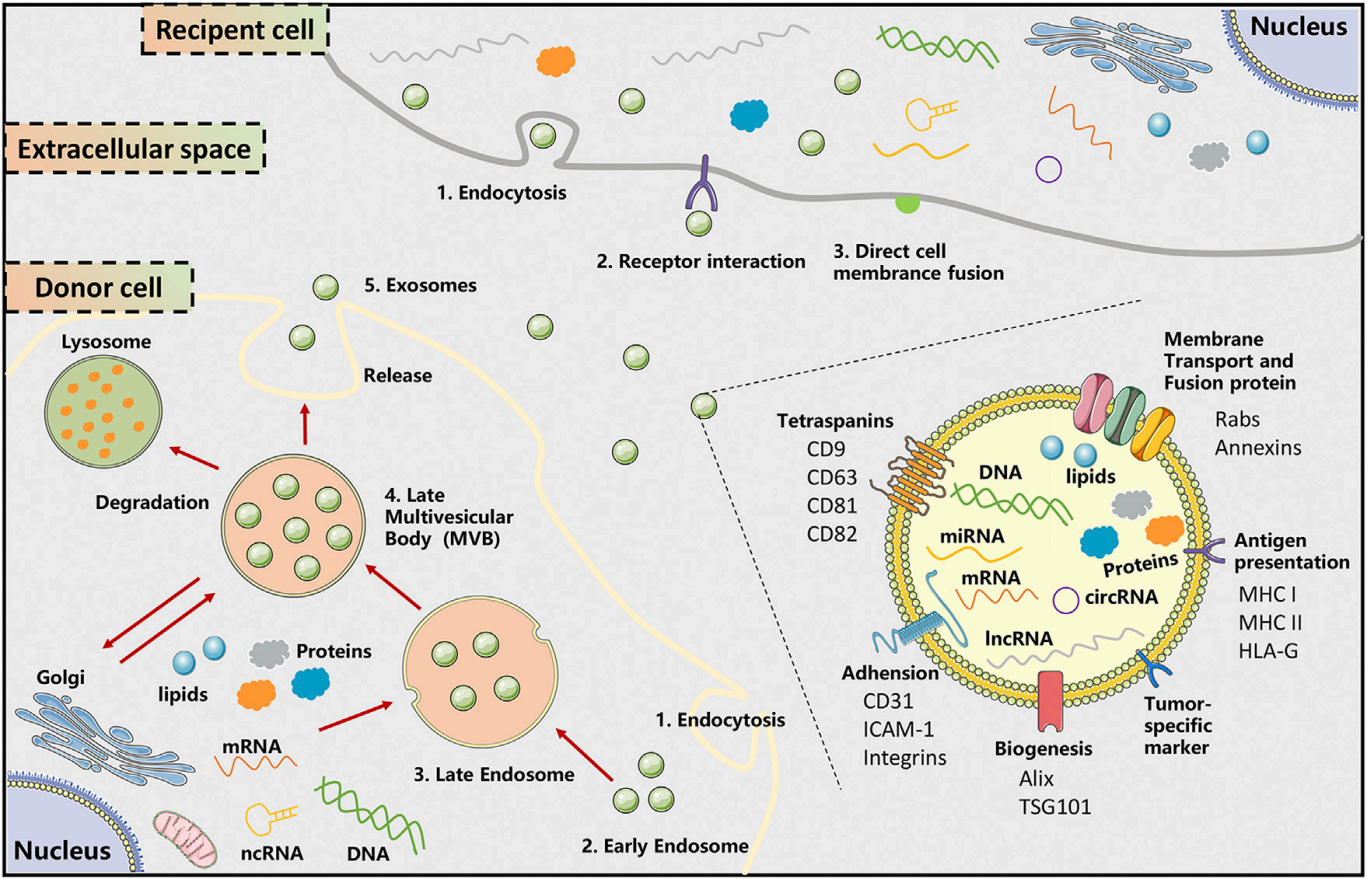
The biogenesis, secretion, and uptake of exosomes. The biogenesis and release of exosomes undergo initiation, endocytosis, multivesicular body (MVB) formation, and exosome secretion. Exosome biogenesis is initiated with the formation of early endosomes that are formed by early endosomes, a process called endocytosis. Then, the incorporation of selected cargos, including multiple proteins, lipids, mRNA, and non-coding nucleic acids, and endosomes resulted in the MVB formation. Finally, SNARE complexes help MVBs fuse with the plasma membrane, releasing intraluminal vesicles into the extracellular space, which is denoted as exosome. These released exosomes can be internalized with recipient cells via direct fusion, endocytosis, and receptor-ligand interactions, leading to a series of cascade events in recipient cells. Exosomes are enriched in a range of conserved proteins, exosome formation-related proteins, and tumor-related proteins. The membrane constituents include tetraspanins (CD9, CD63, CD81, CD82) membrane trafficking and fusion proteins (Rabs, Annexins), adhesion molecular (CD31, ICAM-1, integrins), tumor-specific markers, MHC and HLA-G. The carried cargoes are comprised of multiple mRNA, proteins (enzyme, Hsp70, Hsp 90), ncRNA (miRNA, lncRNA, circRNA), and metabolites. ICAM-1, intercellular adhesion molecule-1; MHC, major histocompatibility complex; MVB, multivesicular bodies.

Extracellular vesicles (EVs) are defined as membrane-bound vesicles released by various cell types under physiological and pathological conditions (Clancy et al., 2015). Generally speaking, based on morphological features, EVs consist of three particles of different sizes in diameter, mainly including exosomes (30–150 nm), micro-vesicles (150–1,000 nm), and apoptotic bodies (500–2,000 nm) (Yiwen Chen et al., 2021). EVs act as a reservoir that encloses a multitude of functional biomolecules, such as lipids, mRNAs, proteins, ncRNA, such as miRNA, long non-coding RNAs (lncRNAs), and circular RNAs (circRNAs) (Kondratov et al., 2020). These contents not only mirror the composition of the donor cell but also the regulated sorting mechanism (Temoche-Diaz et al., 2019). Especially, exosomes are originated via budding from the plasma membrane of cells and undergoing the formation of late endosome and late multivesicular bodies (MVBs). The MVBs traffic to and fuse with the plasma membrane and consequently release exosomes into the extracellular milieu (Urabe et al., 2021). EVs interact with and are internalized by recipient cells via three mechanisms receptor-ligand interactions, direct fusion, and endocytosis (Figure 1). At present, EVs have emerged as key players of intercellular communication by impacting neighboring or distant cells.

The tumor microenvironment (TME) is an inherent heterogeneous entity comprised of multiple tumor cells, the extracellular matrix (ECM), and infiltrating immune cells and vascular cells (Giraldo et al., 2019). EVs are an emerging type of coordinator for regulating cell interactions at local and remote locations of TME. Previous reports have revealed that EV-secreted components are important mediators in modifying the behaviors of migration and invasion of TNBC cells. As early as 2013, O’Brien et al. validated that the exosomes in the serum of TNBC patients significantly increased the invasion of receptor cells (O’Brien et al., 2013). In other words, it meant that EVs from TNBC cells could transfer phenotypic characteristics representing their origin cells to secondary cells. Besides, in a cohort of Italian BC patients, the prevalence of Human Papillomavirus (HPV) DNA in invasive BC subtypes (TN and HER2+) was as high as 44.4% (De Carolis et al., 2019). The most interesting observation was that the serum EVs from BC patients carried the HPV DNA, which could be transmitted to recipient stromal cells via EV mediation and accordingly resulted in the enhanced aggressiveness of the BC epithelial counterpart. This EV transmission process could also be modulated by inflammatory stimuli.

Therefore, EVs have yielded confounding influences on TNBC progression. Tumor-associated EVs can manipulate the metastatic cascade through angiogenesis, signal transduction, drug resistance, genetic intercellular exchange, and pre-metastatic niche formation. More intriguingly, the EV profile of circulation, stability, and particularity in body fluids, might provide clinically reliable biomarkers for TNBC prognostic strategies. Besides, the natural or synthetic EVs could represent suitable carriers for drugs or bioactive molecules to target specific cell populations, posing the EV potential in reversing cancer cachexia. In this review, we will discuss and emphasize the current progression of EVs in the progression, diagnosis, and treatment in the context of TNBC, hoping to decipher the advantages and challenges of EVs for combating TNBC.

## 2 Extracellular Vesicles in Triple-Negative Breast Cancer Growth

With the development of omics technologies, EVs occur as evolving modifiers that lead to dynamic crosstalk in heterogeneous microenvironments. EVs represent a manner for horizontal transfer of neoplastic traits, which could aid tumor growth and exacerbate tumor malignancy. Ozawa et al. confirmed that EVs derived from HCC1806 could strengthen the proliferation and drug resistance ability of non-tumorigenic MCF10A breast cells, accompanied by cancer-related DE miRNA alteration (Ozawa et al., 2018). Sung et al. uncovered an intriguing phenomenon that ITGB4-overexpressing TNBC cells transmitted ITGB4 proteins to cancer-associated fibroblasts (CAFs) in an exosome-independent way, thereby triggering BNIP3L-related mitochondrial autophagy and lactate production in CAFs (Sung et al., 2020). This study indicated a potential therapeutic strategy based on ITGB4-induced mitochondrial autophagy, including blocking the TNBC exosome release, inhibiting the ITGB4-induced JNK activation, and AMPK-mediated mitochondrial autophagy in CAFs. Deoxyelephantopin (DET), the naturally occurring sesquiterpene lactone derived from *Elephantopus scaber*, has been confirmed to possess potent anticancer activity (Duan et al., 2021). The DET and its analogue DETD-35 could suppress the TNBC cell activity by altering the protein composition and function of exosomes and releasing exosomes from cancer cells induced by oxidative stress (Shiau et al., 2017).

The intercellular communication by the EV transmission of ncRNAs reprograms TNBC cell proliferation as oncogenic drivers and tumor suppressors. Ji Eun Kim et al. (2020) detected the exosomal miRNA profile of the cancer-associated fibroblast (CAF) and confirmed a 5-fold decrease in miR-4516 expression compared with normal fibroblast (NF). Moreover, miR-4516 treatment suppressed the CAF-induced promotion of tumor cell proliferation, while the stromal loss of miR-4516 facilitated the FOSL1-dependent proliferation and TNBC malignancy. Xing et al. (2021) showed that the upregulated expression of miR-106a-5p in TNBC neoplastic tissues and TNBC cells was positively related to tumor grade, suggesting a poor prognosis of TNBC patients. Besides, miR-106a-5p packaged in MSC-derived exosomes promoted TNBC tumor progression in the xenograft, which could be suppressed by lncRNA HAND2-AS1. Tong Chen et al. (2021) revealed that circHIF1A, which was upregulated in the plasma of BC patients, could be selectively loaded into exosomes secreted by BC cells. CircHIF1A promoted TNBC growth and metastasis by regulating NFIB expression and subcellular location and was involved in the circHIF1A/NFIB/FUS positive feedback loop.

## 3 Extracellular Vesicles in Triple-Negative Breast Cancer Metastasis

Metastasis is a multistep event that tumor cells experience an evolutionary process, comprising the dissemination from primary sites, intravasation into the lymphatic vessels and blood circulation, and ultimate colonization at distant organs and secondary tumor formation (Yufang Tan et al., 2021). It is well-documented that EVs are crucial participators in conditioning the pre-metastatic niche microenvironment, highlighting the importance of EV biogenesis and constituents in TNBC metastasis (Becker et al., 2016). Here, we conclude that the EV components, mainly including EV-derived proteins and EV-derived ncRNAs, participate in BC metastasis. Especially, EV-derived proteins (cofilin-1, ITG β4, ASPH, UCHL1, SPANXB1, and TGF-β1) and EV-derived ncRNAs (miR-770, miR-9, miR-155, miR-221, miR-939, and circRNA circPSMA1) are the components that have been reported. These factors enter recipient cells through EV uptake pathway and thus enhance the migration, invasion, and metastasis of recipient cells by influencing downstream signaling pathways and a series of cascade reactions. Totally, the evil EV-containing components mediate the reprogram to initiate and promote the BC metastasis. Targeting and educating EV-mediated metastatic alteration are the key clues to effectively block metastasis formation.

### 3.1 Extracellular Vesicle-Derived Proteins in Triple-Negative Breast Cancer Metastasis

The EV biogenesis is a complicated multistep process, while the surface proteome of EVs is a fundamental coordinator that bridges intracellular and extracellular signaling networks (Rai et al., 2021). Howard et al. (2022) revealed that cofilin-1 was an upregulated protein that was associated with grave prognosis in TNBC, and was the most frequently detected component in EVs detected by proteomic investigation. Cofilin-1 was a vital regulator in EV formation and thus potentiated the formation pre-metastatic niche of TNBC cells. Integrin β4 (ITG β4) is a class of transmembrane adhesion molecules associated with the maintenance of cell adhesion (Ruan et al., 2020). Soung et al. (2018) identified that ITG β4+ EVs participated in tumor invasion and were detectable in the cultural supernatants of TNBC cells. The tumor suppressor arrestin domain-containing 3 (ARRDC3) was a key player to inhibit the sorting of ITG β4 into EVs by inhibiting EGF-driven endocytic recycling, thus suppressing the metastatic potentials of EVs.

Tumor-derived EVs participate in organotropic metastasis *via* pre-metastatic niche construction (Mo et al., 2021). Zhang et al. showed that chemotherapy-induced a massive release of phosphatidylserine+ BC cell-derived microparticles (BCMPs), which could be utilized by cancer cells to promote endothelial leakiness and boost metastasis (Zhang et al., 2021). The circulating EVs from BC patients promoted matrix metalloproteinase (MMP)-2 and MMP-9 secretion, migration, and invasion of MDA-MB-231 cells by inducing activation of Src and focal adhesion kinase (Ramirez-Rícardo et al., 2020).

Emerging evidence has indicated that aspartate β-hydroxylase (ASPH) is detectable in advanced/spontaneously metastatic BC but is silenced in normal adult tissues, and is required for the maintenance proliferation, invasion, and metastasis in various cancer types (Zheng et al., 2020). ASPH endowed more malignant behavior in TNBC or Her2, reduced OS/DFS, and early recurrence/progression (Lin et al., 2019). In this process, the ASPH-Notch axis helped to prepare MMPs/ADAMs for exosomes synthesis/release, which was conducive to potentiate multifaceted metastasis. Sipeng Li et al. (2021) identified that the expression level of CD151 in TNBC-derived serum exosomes was markedly higher than that in healthy subject exosomes. Using quantitative proteomics methods, they found that exosome CD151 promoted ribosomal protein secretion through exosomes and restrained exosome complement protein secretion. In addition, the CD151 deficiency in exosomes significantly inhibited the TNBC cell migration and invasion. Liu et al. (2020) found that UCHL1 content was upregulated in the TNBC patient sera, sera exosomes, and TNBC cell conditioned media (CM). Exosomes rich in UCHL1 resulted in BC cell migration and extravasation in a paracrine manner. Specifically, UCHL1 promoted TGFβ-induced metastasis *via* reducing TGFβ1 receptors and SMAD2 ubiquitination. Kannan et al. (2019) emphasized that SPANXB1 was an abundantly expressed cancer-testis antigen in human primary and metastatic TNBC tissues and was detectable in the secreted EVs. Functionally, SPANXB1 acted as an oncogenic promoter that promoted the tumor progression and even the spontaneous metastasis of TNBC, which was possibly mediated by circulating EVs. This study affirmed the uniquely restricted expression of SPANXB1 in TNBC, introducing its potential for TNBC metastasis blocking and prognostication.

Circulating tumor-derived endothelial (TEC)-EVs may provide the soil for cancer cell homing possibly due to their pro-angiogenic properties. Lopatina et al. (2020) showed that the IL-3Rα blockade on TECs could reprogram EVs and alter the EV miR composition, thereby gaining the ability to alter Vimentin, β-catenin, and TWIST1 expression, reducing angiogenesis and lung metastasis of primary tumors *in vivo*. Junyoung Kim et al. (2020) established a three-dimensional (3D) Human Liver-Chip to simulate the premetastatic niche formation by BC-derived EVs. In this system, EV-derived TGF-β1 upregulated the fibronectin in liver sinusoidal endothelial cells (LSECs), leading to enhanced adhesion ability of BC cells. Besides, the TGF-β1 content of blood EVs of patients with BC liver metastasis was significantly higher in comparison to healthy controls and non-metastatic TNBC patients.

### 3.2 Extracellular Vesicle-Derived Non-Coding RNAs in Triple-Negative Breast Cancer Metastasis

The exosome-mediated miR-770 uptake in recipient TNBC cells inhibited the migration and invasion behaviors through the miR-770/STMN1 axis (Li et al., 2018). Kia et al. (2019) showed that miR-9 and miR-155 were one of the overexpressed miRNAs in highly TNBC cells and their exosomes, and could target tumor suppressor gene PTEN and DUSP14 separately. They further utilized the exosomes of highly-metastatic MDA-MB-231 cells to treat low-metastatic MCF-7 cells, leading to the downregulation of PTEN and DUSP14 in recipients and increased tumor metastasis *in vitro*. Additionally, the PAR2-derived MVs of MDA-MB-231 cells conferred the pro-tumorigenic epithelial to mesenchymal transition (EMT) and metastasis phenotypes of recipient cells by transferring miR-221 in a PTEN/AKT/NF-ĸB/miR221 dependent manner (Das et al., 2019). Di Modica et al. (2017) presented that miR-939 expression in TNBC tissues was associated with poor prognosis, and the exosomal miR-939 secreted by TNBC cells to downregulate VE-cadherin and destroy the barrier function of endothelial monolayers. This result posed an extracellular pro-tumorigenic role of exosomal miR-939 in regard to blood vessel invasion. Su-jin Yang et al. (2021) confirmed that circRNA circPSMA1 was overexpressed in TNBC cells, as well as the exosome from tumor cells and serum from TNBC patients. By exosome-mediated transmission pathway, circPSMA1 could promote tumorigenesis, metastasis, and immunosuppression state by regulating the circPSMA1/miR-637/Akt1-β-catenin (cyclin D1) axis in TNBC both *in vitro* and *in vivo*.

## 4 Extracellular Vesicles in Triple-Negative Breast Cancer Immune Regulation

TNBC is a potential protagonist of immunotherapy in BC manifested by diverse immune signatures with abundant tumor-infiltrating lymphocytes, multifaceted functional immune factors, and immunosuppressive surveillance (Razazan and Behravan, 2020). EVs function as significant mediators of immune regulation in cancer. EVs released from tumor cells, stromal cells and activated immune cells exert pleiotropic effects on immune strength against cancer. More importantly, it has been theorized that reversal or recovery of anti-tumor immune response is fundamental for halting the tumor metastasis and enhancing curative effects in TNBC (Brunner, 2019). The plethora of signals conveyed by EVs can either support or restrain the immunosuppression of lymphoid (T lymphocytes, B lymphocytes, and NK cells) and myeloid (macrophages, dendritic cells, monocytes, myeloid-derived suppressor cells, and neutrophils) cell populations in tumors (Kugeratski and Kalluri, 2021).

The role of BC cell-derived EVs in fostering macrophage polarization is a fascinating issue in TNBC progression. Rabe et al. (2021) demonstrated that tumor EVs were able to educate non-resident naïve macrophages to pro-metastatic TAM phenotype in TNBCs. In terms of mechanism, tumor-expressing CCL5 was the key mediator in regulating the biogenesis, secretion, cargos of EVs, as well as shaping the secretion profile of macrophages. Piao et al. (2018) suggested that BC-derived exosomes stimulated the macrophage polarization, characterized by an increased ratio of M2/M1 polarized *in vitro* and in an orthotopic model, which established suitable pro-metastatic conditions for axillary lymph node (LN) metastatic processes in TNBC. The EVs protein content modulation cooperated with enhanced TGF-β and IL-17F secretion, was involved in M2 polarization and Prune-1-promoted lung metastasis in the mouse TNBC model (Ferrucci et al., 2021).

It is believed that limited infiltration and inhibition activity of specific T cells in the immunosuppressive TME are recognized to be important obstacles in cancer immunotherapy. Programmed death-1 (PD-1) is an immune checkpoint expressed on activated T cells and acts as a pivotal tumor suppressor in T cell lymphomas, through binding programmed death-ligand 1 (PD-L1) (Wartewig and Ruland, 2019). Immunotherapy based on PD-1/PD-L1 has been proved to be a promising option to treat hematologic malignancies, as well as solid malignant tumor types (Cherkassky et al., 2016). Notably, the inhibitory signals triggered *via* the PD-1/PD-L1 pathway are critical to balancing the activation, tolerance, and exhaustion of T cells (Catherine L. Tan et al., 2021). Nevertheless, it is a conflicting issue that the higher PD-1 expression in tumor-infiltrating lymphocytes (TILs) is markedly related to better survival in TNBC patients. Qiu et al. (2021) found that exosomal PD-1 from activated T cells resulted in enhanced immune surveillance by neutralizing PD-L1/PD-1-cooperative cell-killing capacity of tumor-infiltrating CTLs in the TNBC mouse model. In addition to these, there is a close association between BC tissue and mechanical property-induced biological changes within a complex and three-dimensional TME. Wang et al. (2020) posing an important point that exposure to mechanical strain, such as oscillatory strain, could promote the PD-L1+ exosome release and BC proliferation. The BC-derived exosome could be internalized and localized in immune cells and stromal cells in TME, and then enriched the abundance of myeloid-derived suppressor cells (MDSCs) and M2 macrophages and reduced the CD8^+^ T cells. It manifested that mechanical strain was an important factor that potentiated the malignant EMT and metastasis behavior and enhanced the release of immunomodulatory exosomes in BC.

Validating whether these exosomal immune factors are co-packaged in single or accurately encapsulated into different exosome populations, would help illustrate the complicated roles of T cell-derived exosomes. It also provides a feasible strategy by modifying the burden of surface inhibitory immune checkpoint (ICP) receptors loaded in exosomes, such as PD-1, Tim-3, and LAG-3, in combination with conventional chemoradiotherapy methods, for the reinforcement of the immune killing effect. Yet, up to now, the quantification and specific intracellular regulation of immunogenic molecules in/on exosomes in TNBC remain largely unknown. Together, these studies propose a potential perspective on manipulating EVs to boost immune responses for better TNBC outcomes.

## 5 Extracellular Vesicles in Triple-Negative Breast Cancer Diagnosis

Many TNBC patients are diagnosed at an advanced stage, partly because of a lack of precise biomarkers. The identification of valuable biomarkers is urgently needed for early detection and therapy of cancers, especially for high-risk patients. Conceptually, tissue biopsy is widely perceived as the gold standard for TNBC diagnosis. At present, EV-based liquid biopsies are gaining momentum in clinical research in BC. EVs are extensively and stable existed in biological fluids, including serum, plasma, urine, saliva, and ascites, and can therefore be isolated for clinical evaluation (Yu et al., 2021). Especially, tumor-derived EVs carry multiple types of bioactive molecules, including DNA, mRNAs, ncRNAs, enzymes lipids, and metabolites. Thus, EVs and their characteristic compositions are potential platforms for early detection, prognosis evaluation, and recurrence monitoring in TNBC.

### 5.1 Extracellular Vesicle-Derived Proteins in Triple-Negative Breast Cancer Diagnosis

In the clinic, some specific membrane markers and packaged proteins in TNBC cell-derived EVs, are estimable signatures for non-invasive diagnosis and prognosis of multiple cancers. Yoh et al. (2021) collected the EpCAM+ EVs from the plasma of patients with non-small cell lung carcinoma (NSCLC) and TNBC. They subsequently confirmed that the heterogeneous EpCAM+ EVs were originated from tumor sites and demonstrated the clinical diagnostic potential value of cancer-EVs specific expressed biomarker, such as PD-L1. In the cohort of American women, Chaudhary et al. (2020) investigated the relationship of serum exosomal-annexin A2 (exo-AnxA2) and clinicopathological features of BC patients, showing that the higher expression of exo-AnxA2 levels indicated more serious tumor grade, poorer overall survival (OS), and disease-free survival (DFS). Besides, the exo-AnxA2 expression in African-American (AA) women with TNBC was significantly higher than that of Caucasian American TNBC. These results sent a message that the exo-AnxA2 possessed an excellent area under curve (AUC) value in evaluating TNBC aggression features.

Drug resistance is considered to be a major concern in the successful treatment of cancer. The proposed biomarkers and methods for predicting therapeutic resistance will offer enormous value in drug implementation (Milman et al., 2019). In the TNBC tissues, there was a significant association between annexin A6 (ANXA6) and EGFR expression (Ting Li et al., 2021). When receiving gemcitabine-based first-line chemotherapy, the serum levels of exosomal ANXA6 were lower in highly sensitive TNBC patients than in resistant TNBC patients, demonstrating that ANXA6 in serum exosome was a robust indicator for predicting the response to gemcitabine-based chemotherapy (Ting Li et al., 2021).

### 5.2 Extracellular Vesicle-Derived Non-Coding RNAs in Triple-Negative Breast Cancer Diagnosis

EV-derived ncRNAs represented by miRNA, lncRNA, and circRNA, among which tumor-specific miRNA are the most studied components. miRNAs are attractive tools in regulating cell signaling, homeostasis, and fate by acting as tumor suppressors or oncogenes (Rupaimoole and Slack, 2017). There is an aberrant expression pattern in single miRNA or miRNAs signature in TNBC-derived EVs. Wu et al. (2020) screened out a total of 20 upregulated and 34 downregulated miRNAs that were DE miRNAs in plasma exosomes of TNBC and healthy controls. The upregulated miR-150-5p and miR-4665-5p were of the capability to distinguish BC with recurrence and non-recurrent BC patients, which might be conducive to preventive strategies for BC. Ozawa et al. (2020) isolated the serum EVs of healthy controls and patients with luminal A and TNBC in Brazil, and accordingly identified the expression of 4 EV-miRNAs, including miR-142-5p, miR-150-5p, miR-320a, and miR-4433b-5p. The miRNA profile comprising miR-142-5p, miR-320a, and miR-4433b-5p distinguished BC patients from healthy controls with a sensitivity of 93.33% and specificity of 68.75%, while the decreased expression pattern of miR-142-5p and miR-150-5p indicated a more advanced stage of tumor classification.

In clinical practice, some other studies reported provide perspectives into the clinical application of serum EV-miRNAs as specific biomarkers for predicting the effectiveness of different containment strategies in TNBC. In a randomized phase II neoadjuvant GeparSixto trial, Stevic et al. (2018) confirmed that a total of 17 miRNAs in the TNBC subgroup and 10 miRNAs in the entire cohort of BC patients were observably deregulated. Among them, miR-155 and miR-301 were the optimal candidates for predicting pathological complete response (pCR) separately in the univariate and multifactorial analysis. It indicated that the differentially expressed exosomal miRNA characteristically distinguished different tumor subtypes, reflected different stages, and was related to risk factors, and might be promising monitoring biomarkers for BC. Salvador-Coloma et al. (2020) confirmed the potential of circulating exosomal miRNA signature in predicting neoadjuvant chemotherapy (NAC) response in TNBC, which targeted the pathways of immune maturation and was related to immune suppression. The expression levels of miR-185, miR-4283, miR-5008, and miR-3613 were lower in patients without response, and the expression levels of miR-1302, miR-4715, and miR-3144 were higher.

LncRNAs, which are vital components of the genetic program that changes tumor cell-intrinsic properties, have drawn increasing attention as crucial players in mediating tumor initiation and progression (Hu et al., 2020). As the lncRNA transcript panel is associated with TNBC, ER+ subtypes, and normal breast tissue, the molecular classification of BC based on lncRNA transcriptome characteristics could efficiently identify novel diagnostic lncRNA signature for TNBC (Rodríguez Bautista et al., 2018). The SUMO1P3 is an abnormally expressed lncRNA in multiple cancers. Especially, the upregulation of serum exosome SUMO1P3 was robustly associated with an unfavorable prognosis and treatment failure in TNBC, in comparison to the other patients with benign diseases or healthy controls (NA-ER et al., 2021). Lan et al. (2021) revealed that the serum exosome lncRNA XIST was significantly decreased after primary breast tumor resection, but increased significantly after recurrence in TNBC. This study posed that the serum exosome lncRNA XIST level could serve as an efficient assessment tool to predict recurrent TNBC load status, independent of other clinicopathological parameters.

## 6 Extracellular Vesicles in Triple-Negative Breast Cancer Treatment

The existing studies of EVs in TNBC treatment mainly focus on drug resistance and drug carriers. The cancer-derived EVs can support the drug-resistant phenotypes via acting in a paracrine and systemic manner, thus leading to reduced efficacy and treatment failure (Xavier et al., 2020). EVs have an impact on *de novo* and acquired resistance bioprocesses. Besides, as natural nanoparticles, EVs possess unique excellent homing propensity, immune compatibility, low toxicity, and conservatively enclosed structure, thus conferring the ability to utilize EVs to encapsulate diversified small molecule drugs or biological macromolecules (Vasconcelos et al., 2019).

### 6.1 Extracellular Vesicles in Drug Resistance of Triple-Negative Breast Cancer

The novel targeted agents, including PARP inhibitors, antibody-drug conjugates, and immune-checkpoint inhibitors, have been devoted to TNBC treatments (Mehanna et al., 2019). The patients with TNBCs exhibit frequent relapsed and metastatic features and are still confronted with adverse resistance to chemotherapy and radiotherapy. MDA-MB-231 cell-derived MVs imparted the cisplatin resistance of MCF-7 by transferring MV miR-221, which could be negated by miR-221 inhibition (Das et al., 2019). Wang et al. (2019) the exosomal miR-423-5p secreted by cisplatin-resistant MDA-MB-231 cells, promoted the therapeutic sensitivity for cisplatin in recipient cells, manifesting as enhanced IC_50_ and reduced apoptosis ratio. Clinical evidence showed that miR-770 was aberrantly upregulated in chemo-sensitive tissue of TNBC (Li et al., 2018). The further *in vitro* and *in vivo* assay confirmed that exosomal miR-770 suppressed the doxorubicin-resistance via regulating apoptosis and TME by targeting STMN1 mRNA.

In TNBC patients treated with first-line gemcitabine-based chemotherapy, the highly sensitive patient exhibited a higher expression lever of ANXA6 compared with low sensitive patients (Ting Li et al., 2021). Besides, exosomes derived from drug-resistant BC cells improved the gemcitabine resistance by transferring ANXA6, which was strongly related to the inhibition of EGFR ubiquitination and degradation. Therefore, serum exosome level of ANXA6 may be a robust predictor of gemcitabine response to chemotherapy for TNBC patients. The gemcitabine-resistant cancer cells could release exosomes to enhance the proliferative capacity and suppress the apoptosis of sensitive cancer cells by delivering exosomal ANXA6, which was closely associated with the downregulation of EGFR ubiquitination and degradation (Ting Li et al., 2021). It was endowed with the potential of serving as a promising alternative treatment for chemoresistant mTNBC by inhibiting the function of exosomal ANXA6 or EGFR.

### 6.2 Extracellular Vesicles as Drug Carriers in Triple-Negative Breast Cancer Therapy

The genetically engineered T cells with a chimeric antigen receptor (CAR) expression have emerged as a distinctively promising therapy for hematological and solid malignancies (Hong et al., 2020). The biomimetic nanovesicle CAR-containing exosomes contain a series of high-level cytotoxic molecules (Fu et al., 2019). CAR-T cell-derived exosomes represent a preferred delivery platform for magnifying the immune killing effect. Yang et al. found that the exosome derived from the mesothelin-targeting CAR-T cells, both retained the characteristics and surface expression of their parental CAR-T cells and effectively killed the tumor by secreting perforin and granzyme B, accompanied with a favorable safety profile (Pengxiang Yang et al., 2021).

The EVs derived from mesenchymal stromal cells (MSCs) have been broadly investigated as in various drug delivery systems for cell-free therapy, possessing the natural ability of homing to BC tumor, low immunogenicity, and elastic properties (Pinky et al., 2021). The EVs isolated from human umbilical cord mesenchymal stem cells (hUCMSCs) could upload cannabidiol (CBD) to increase the doxorubicin (DOX) sensitivity of MDA-MB-231 cells, leading to increased apoptosis expression and reduced tumor burden (Patel et al., 2021). EV derived from adipose tissue-mesenchymal stromal cells (ADSCs), could mediate the transmission of miR-424-5p to inhibit the PD-L1/PD-1 signaling, thus conferring an inflammatory TME and enhancing immunotherapy (Patel et al., 2021). Shojaei et al. showed that the miR-381-carrying ADSC-exosomes could be internalized by MDA-MB-231 cells, resulting in inhibited proliferation, migration, and invasion by altering EMT-related gene expression (Shojaei et al., 2021).

EVs can be utilized as efficient transport carriers with the advantages of design flexibility, functionalized modification, targeted delivery, and low cytotoxicity in an integrated systematic mode (Alghuthaymi et al., 2021). Li et al. (2020) reported a nanoplatform comprising engineered macrophage exosomes, c-Met-targeting peptide, and DOX, which exhibited an excellent antitumor effect in TNBC xenograft. Yu et al. (2019) established folate-vectorized exosome system (Erastin@FA-exo), which could improve the targeting and biocompatibility of this formulation to selectively and efficiently induce ferroptosis in TNBC cells. Gong et al. (2019) fabricated a synergistic delivering carrier based on targeted exosome *via* biomimicry pathway. This nano-carrier efficiently uploaded Dox and miRNA-159 and resulted in an enlarged anti-cancer effect in TNBC therapy. Zhao et al. (2020) successfully constructed a self-assembled nanoparticle based on cationic bovine serum albumin (CBSA) conjugated siS100A4 and exosome, which could heighten the suppression of postoperative lung metastasis in the TNBC model. The exosome was derived from the packaged autologous exosome membranes with the capability of delivery and minimized immunogenicity.

## 7 Limitations and Perspectives

Nowadays, BC is a very high incidence and malignant female tumor type, and EVs are an important target for tumor-related mechanisms, diagnosis, and treatment. Therefore, the function and role of EVs in BC are a major focus of current research. There are many subtypes of BC, including ER+ and TNBC, but the existing studies are mainly focused on the broad spectrum of BC, that is, most of the research has not focused on specific subtypes of BC. The components of EVs are relatively complex, mainly including exosomes and MVs, but the main components that perform such functions are exosomes. In general, EVs are involved in all aspects of BC, including tumor initiation, malignant growth, metastasis (tumor colonization, lymphatic and blood metastasis, and organ colonization), early diagnosis, prognostic monitoring, and drug resistance monitoring. Besides, as a typical TNBC cell line, the exosome secreted by MDA-MB-231 is the most commonly reported type. As mentioned above, we have provided a preliminary landscape of EVs in TNBC. However, there still are certain critical challenges deserving our attention in this field, including standardized processed EV methods, thorough oncological mechanism, diagnostic efficiency, and therapeutic applications.

Firstly, for EV obtainment, many methods for separating EVs have been intensively improved over the last decade, but no standardized method for characterization of single EVs in various studies. There are still obstacles in the establishment of the standard methods of EV isolation, acquirement, and characterization analysis. The standardization of different processes, from EV isolation to storage conditions, is beneficial to avoid variability across different studies. This will help interpret data from different tumor models, treatments, and research executors. Secondly, in the aspect of diagnosis capability, due to different sample resources, screening criteria, and processing methods, the same index varies in different diagnostic studies, which will lead to inconsistent sensitivity and detection limits. There may also be other cell fragments, impurities, or vesicles of similar size that interfere with biomarker detection. The optimization of methodologies is necessary to characterize the comprehensive profile of encapsulated molecules in EVs.

Thirdly, the oncological mechanisms of EVs are relatively inadequate in TNBC. The organotypic metastasis caused by tumor-derived EVs is the most fetal performance in TNBC and needs deepgoing elucidation. Cancer cells are now considered to produce more amount of EVs than their non-malignant counterparts. Cancer cell-derived EV-related epigenetic alterations, represented by methylation, histone modification, and especially ncRNA. In addition to miRNA, the role of EV-epigenetic regulation in TNBC has not been extensively and deeply explored. Besides, the role and possible alteration of immune regulation in establishing pre-metastatic niches in metastatic organs need further investigation. The BC TME is a complex entity composed of a variety of cell types. These cells can remodel the BC progression through regulatory mechanisms such as autocrine and self-differentiation. Moreover, the sustained growth and colonization of TNBC cells require bidirectional cell-cell communication predominantly involved in stroma cells, endothelial cells, and local immune cells, indicating an intricate interaction network. Due to this complexity, it is not clear which or which cell-derived EV components play a crucial regulatory role in a given situation. Therefore, in many studies, it is generally focused on a specific exosome target, rather than multiple targets. Accurate tracking of the location, distribution, and ultimate fate of EVs is also crucial to exploring the internalization and goals of EVs. In general, gene knockout, cell co-culture, EV staining tracer, suborganelle co-location, and *in vivo* imaging are used to determine the occurrence, transport, uptake, and biological effects of EV on recipient cells. Once EVs are ingested by recipient cells, specific components of the exosomes, along with other key molecules, trigger a cascade of effects that lead to changes in recipient cell behavior that support or arrest tumor progression.

For future perspectives, EVs are promising candidates as biomarkers for BC diagnosis and therapy. Circulating nucleic acids and proteins, encapsulated within small EVs, provide a realistic photograph of cancer features. EV components are reported to accurately discriminate TNBC samples from controls and estimate the progression and prognosis. Hence, EV detection would bring to the forefront transformational diagnostic strategies. Unfortunately, due to the lack of relevant studies and the complex heterogeneity of breast tumors, the diagnosis of TNBC by exosomes is still superficial. There are no relevant reports to show that exosomes and related contents have been used to guide clinical drug monitoring or to build predictive models. Furthermore, it is currently a difficult problem to effectively separate circulating EVs and achieve sufficient sensitivity and accuracy for discrimination. With next-generation sequencing advancement, the ongoing excavation of novel reliable biomarkers or signatures of EVs, is undoubtedly conducive to predicting the prognosis and management of the patients with TNBC. The EV detection application combined with other clinical routine indicators, such as clinicopathological parameters and other *in vitro* detection (IVD), will usher in a promising, precise, and individualized diagnostic mode. Therefore, the deeper and more systematic evaluations based on large randomized-control trials are still urgent to fully define the potency of EV biomarkers.

Lastly, the naturally occurring and technically modified EVs are emerging candidates as anti-cancer drug delivery vehicles, with the hope of achieving maximum efficacy and minimal side effects in TNBC drugs or gene therapy. EVs possess a stable structure, good stability, good carrier properties, and low immunogenicity, making them suitable for biological drug delivery. Many studies now use TNBC cells as model cells or tumor-bearing mouse models to conduct application studies related to EV drug loading. How to effectively upload drugs or conduct gene function transduction is a key point at present. Optimizing drug loading methods for EVs is a prerequisite for subsequent therapeutic applications. Besides, there is an interesting paradigm in EV biology that blocking cancer cell-derived EV biogenesis, release, uptake, and/or the uploaded key components, might add another weapon for cancer treatment alone or in conjunction with conventional therapies (Xu et al., 2018). This is due to the intervention of EVs-mediated tumor signaling for favoring tumor evolution. The EVs derived from immune cells, such as macrophage, NK, and CAR-T, are excellent candidates for eliciting endogenous antitumor immunity. These EVs could be applied either singly or in combination for treating TNBC. But these are all in the very early stage of research, basically staying at the level of cell and animal research. Further tailored clinical studies on large scales are now warranted to validate these thoughtfully transformed EV carriers.

## 8 Conclusion

**FIGURE 2:**
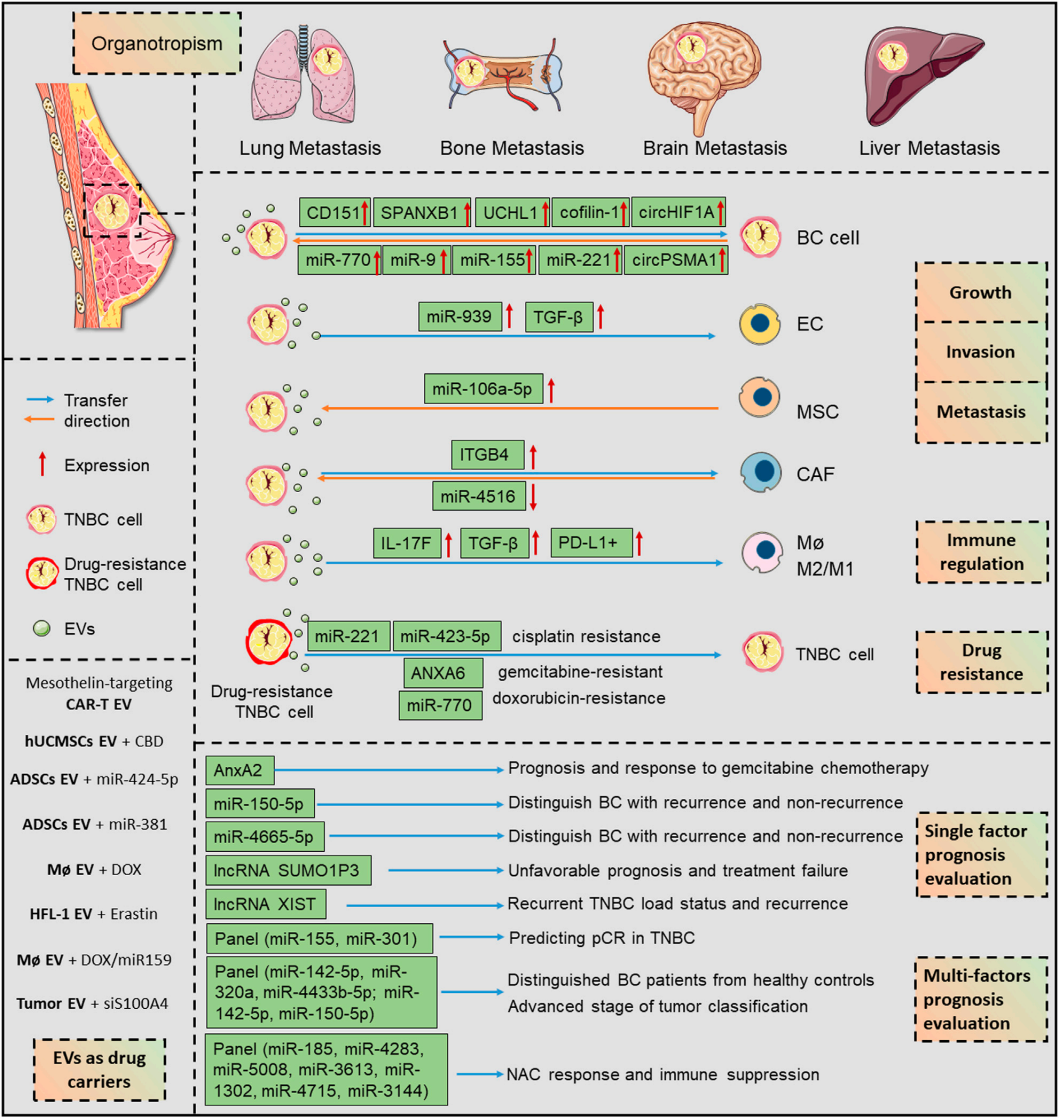
The EV-associated mechanisms in TNBC progression and their potential applications in diagnosis and treatment. EVs evoke numerous pathways that are intensively associated with growth, invasion, and metastasis, during their bidirectional interactions with multiple cell types in tumor environment. The horizontal transfer of EV cargoes, represented by protein, miRNA, lncRNA, circRNA, are depicted as important orchestrators in shaping the TNBC progression. Besides, EVs are as promising biomarkers for TNBC state, prognosis, and therapeutic effect evaluation. Due to their stable carrier properties, EVs are promising engineered nano-carriers for encapsulating biological compounds for TNBC therapy. ANXA6, annexin A6; BC, breast cancer; CAF, cancer-associated fibroblasts; CAR-T, chimeric antigen receptor T cell; CBD, cannabidiol; DOX, doxorubicin; EC, endothelial cell; EV, extracellular vesicle; ITGB4, integrin β4; lncRNA, long non-coding RNA; M2/M1, alternative anti-inflammatory activation (M2), pro-inflammatory activation (M1) and; Mø, macrophage; MSC, mesenchymal stromal cells; NAC, neoadjuvant chemotherapy; pCR, pathological complete response; TNBC, triple-negative breast cancer.

Collectively, the horizontal propagation of EVs could modulate intercellular communication in TME, leading to malignant growth, metastasis, and drug resistance of TNBC *via* multiple molecular mechanisms (Figure 2; Table 1). EVs are revolutionizing the diagnostic and therapeutic landscape of TNBC, owing to their distinctive content of proteins and genetic materials that mirror the cellular origin. Although compelling studies have confirmed that EVs contribute to the TME plasticity and multiple stages of TNBC progression, experimental evidence and mechanical elaboration to define EVs as important regulators are only beginning. Further investigation will allow personalized EV-based intervention for combating TNBC.

**TABLE 1:** EVs and their constituents in remodeling TNBC behaviors.

EV constituents	Expression patterns	Donor/Recipient cells	Roles and mechanisms	Clinic values	Ref
EVs	Upregulated	HCC1806 cells/MCF10A breast cells	Strengthened the proliferation and drug resistance ability	Potential therapeutic targets	Ozawa et al. (2018)
Exosomes ITGB4	Overexpressing	TNBC cells/CAFs	Triggered BNIP3L-related mitochondrial autophagy and lactate production in CAFs, promote TNBC	ITGB4-induced mitophagy as targeted therapy	Sung et al. (2020)
Exosomes	Downregulated by DET/DETD-35	MDA-MB-231/Homologous cells	DET/DETD-35 inhibited TNBC cell activity through ROS-mediated exosomal activity and protein functions	DETD-35 as a potential anti-TNBC drug	Shiau et al. (2017)
Exosomes miR-4516	Upregulated	CAFs/TNBC cells	The stromal loss of miR-4516 facilitated the FOSL1-dependent proliferation and TNBC malignancy	miR-4516 as an anti-cancer drug for TNBC	Ji Eun Kim et al. (2020)
Exosomes miR-106a-5p	Upregulated	MSCs/TNBC cells	miR-106a-5p packaged in MSC-derived exosomes promoted TNBC tumor progression in xenograft	An attractive target in TNBC treatment	Xing et al. (2021)
Exosomes circHIF1A	Upregulated	MDA-MB-231/Homologous cells	CircHIF1A promoted TNBC growth and metastasis, and was involved in circHIF1A/NFIB/FUS positive feedback loop	A potential therapeutic target for TNBC treatment	Tong Chen et al. (2021)
EVs cofilin-1	Upregulated	Parent cells	Functioned as a vital regulator in EV formation and thus potentiated the formation of the pre-metastatic niche	Target cofilin-1 for the treatment of metastatic TNBC	Howard et al. (2022)
EVs ITG β4	Upregulated	MDA-MB-231/Homologous cells	Downregulation of ITG β4+ EVs by ARRDC3 reduced invasive potentials of TNBC EVs	Therapeutic targeting of ARRDC3/ITG β4 pathway	Soung et al. (2018)
EVs	Upregulated	BC blood EVs/MDA-MB-231	Induced migration and invasion *via* a Src-dependent pathway in TNBC	Potential therapeutic targets	Ramirez-Rícardo et al. (2020)
Exosomes CD151	Enriched in TNBC-derived serum exosomes	231-CD151KO-Exo/MDA-MB-468 and MDA-MB-231	CD151-deleted exosomes significantly decreased the migration and invasion of TNBC cells	Exosomal CD151 as a potential therapeutic target for TNBC	Sipeng Li et al. (2021)
Exosomes UCHL1	Upregulated	MDA-MB-436	UCHL1 as a candidate oncoprotein that promoted TGFβ-induced BC metastasis by protecting TβRI and SMAD2 from ubiquitination	UCHL1 as a potential target for TNBC treatment	Liu et al. (2020)
EVs SPANXB1	Upregulated in circulating sEVs	MCF-7 cells/Homologous cells	SPANXB1 depletion prevented TNBC progression through augmented SH3GL2 expression	Potential for TNBC metastasis blocking and prognostication	Kannan et al. (2019)
EVs IL-3R	Highly expressed in TNBC cells	TEC-EV/MDA-MB-231 cells	Anti-IL-3R-EVs and antago-miR-24-3p-EVs upregulated SPRY2 in MDA-MB-231 cells	Anti-IL-3R-EV for TNBC therapy	Lopatina et al. (2020)
Exosomes miR-770	Highly expressed in chemo-sensitive tissues	TNBC cells/Homologous cells	Inhibited the migration and invasion behaviors through the miR-770/STMN1 axis	A new marker for TNBC chemo-resistance and metastasis	Li et al. (2018)
Exosomes miR-9 miR-155	Overexpressed	MDA-MB-231 cells/MCF-7 cells	MiR-9 and miR-155 were enriched in metastatic BC exosomes targeting PTEN and DUSP14	Novel therapy methods for TNBC metastasis	kia et al. (2019)
MVs miR-221	Overexpressed	MDA-MB-231 cells/Homologous cells	Promoted EMT and metastasis phenotypes in a PTEN/AKT/NF-ĸB/miR221 dependent manner	A potential therapeutic approach for tumorigenesis and metastasis	Das et al. (2019)
Exosomes miR-939	Upregulated	MDA-MB-231 cells/endothelial cells	Downregulated VE-cadherin and destroyed the barrier function of endothelial monolayers	A potential therapeutic target for TNBC invasion	Di Modica et al. (2017)
Exosomes circPSMA1	Overexpressed	TNBC cell lines/Homologous cells	Promoted tumorigenesis, metastasis, and immunosuppression state by regulating the circPSMA1/miR-637/Akt axis	A potential target for TNBC treatment	Su-jin Yang et al. (2021)

CAFs, cancer-associated fibroblast; DET, deoxyelephantopin; DETD-35, DET derivative-35; EMT, epithelial to mesenchymal transition; EVs, extracellular vesicles; ITGB4, integrin beta 4; MSCs, mesenchymal stem cells; ROS, reactive oxygen species; TNBC, triple-negative breast cancer; TEC, tumor-endothelial cells.

## Glossary

AT-MSCs: adipose tissue-mesenchymal stromal cells
AA: African-American
AUC: area under curve
ASPH: aspartate β-hydroxylase
BC: breast cancer
BCMPs: breast cancer cell-derived microparticles
CAFs: cancer-associated fibroblasts
CBD: cannabidiol
CBSA: cationic bovine serum albumin
CAR: chimeric antigen receptor
circRNAs: circular RNAs
CM: conditioned media
DET: deoxyelephantopin
DFS: disease-free survival
DOX: doxorubicin
ER: estrogen receptor
exo-AnxA2: exosomal-annexin A2
ECM: extracellular matrix
EVs: extracellular vesicles
Erastin@FA-exo: folate-vectorized exosome system
HER-2: human epidermal growth factor receptor 2
HPV: Human Papillomavirus
hUCMSCs: human umbilical cord mesenchymal stem cells
ICP: immune checkpoint
IVD: *in vitro* detection
ITG β4: integrin β4
LSECs: liver sinusoidal endothelial cells
lncRNAs: long non-coding RNAs
MMP: matrix metalloproteinase
MSCs: mesenchymal stromal cells
MVBs: multivesicular bodies
MDSCs: myeloid-derived suppressor cells
NAC: neoadjuvant chemotherapy
NSCLC: non-small cell lung carcinoma
pCR: pathological complete response
PARPs: poly(ADP-ribose) polymerases
OS: poorer overall survival
PR: progesterone receptor
TNBC: triple-negative breast cancer
TME: tumor microenvironment
ARRDC3: tumor suppressor arrestin domain-containing 3
TEC: tumor-derived endothelial
